# Renal and Urinary Levels of Endothelial Protein C Receptor Correlate with Acute Renal Allograft Rejection

**DOI:** 10.1371/journal.pone.0064994

**Published:** 2013-05-22

**Authors:** Lionel Lattenist, Jesper Kers, Nike Claessen, Ineke J. M. ten Berge, Frederike J. Bemelman, Sandrine Florquin, Joris J. T. H. Roelofs

**Affiliations:** 1 Department of Pathology, Academic Medical Center, University of Amsterdam, Amsterdam, The Netherlands; 2 Renal Transplant Unit, Department of Nephrology, Academic Medical Center, University of Amsterdam, Amsterdam, The Netherlands; 3 Department of Pathology, Radboud University Nijmegen Medical Center, Nijmegen, The Netherlands; Universidade de Sao Paulo, Brazil

## Abstract

The Endothelial Protein C Receptor (EPCR) is expressed on leukocytes, on endothelium of large blood vessels and to a lesser extent on capillaries. Membrane bound EPCR plays an important role in the activation of protein C which has anticoagulant, anti-inflammatory and cytoprotective effects. After cleavage by a protease EPCR is also found as a soluble protein. Acute rejection of kidney allografts can be divided in T-cell-mediated rejection (TCMR) and antibody-mediated (ABMR) rejection. The latter is characterized by strong activation of coagulation. Currently no reliable non-invasive biomarkers are available to monitor rejection.

Renal biopsies were available from 81 renal transplant patients (33 without rejection, 26 TCMR and 22 ABMR), we had access to mRNA material, matched plasma and urine samples for a portion of this cohort. Renal EPCR expression was assessed by RT-PCR and immunostaining. Plasma and urine sEPCR levels were measured by ELISA.

ABMR patients showed higher levels of EPCR mRNA than TCMR patients. EPCR expression on glomeruli was significantly elevated in ABMR patients than in TCMR or control patients. In the peritubular capillaries EPCR expression was higher in ABMR patients than in control patients. EPCR expression was higher in tubules and arteries of rejection patients than in control patients. Plasma sEPCR levels did not differ. Urine sEPCR levels were more elevated in the ABMR group than in patients with TCMR or without rejection. ROC analysis demonstrated that urinary sEPCR is appropriate to discriminate between ABMR patients and TCMR or control patients. We conclude that urinary sEPCR could be a novel non-invasive biomarker of antibody mediated rejection in renal transplantation.

## Introduction

The Endothelial Protein C Receptor (EPCR) is a type 1 transmembrane glycoprotein which belongs to the CD1 receptor family[1]. EPCR is primarily expressed on monocytes[2], neutrophils[3], the endothelium of large blood vessels and to a lesser extent on capillaries[4]. It binds to the Gla domain of Protein C (PC)[5] resulting in a 20 fold increase of the PC activation rate[6]. Once activated, Active Protein C (APC) can be released in the circulation or can stay bounded to EPCR. Circulating APC plays an important role as an anticoagulant by proteolytically degrading the coagulation factors Va and VIIIa[7], which are important co-factors in the intrinsic and common pathways of the coagulation cascade. EPCR bound APC signals through the G protein-coupled Protease-Activated Receptor 1 (PAR-1)[8] and exerts several anti-inflammatory and cytoprotective effects such as inhibition of the release of inflammatory mediators[9], [10], [11], [12], down-regulation of the expression of adhesion molecules[13], inhibition of neutrophil and eosinophil migration[3], [14], anti-apoptotic activities[13], [15] and the protection of endothelial barrier function[16], [17].

The soluble form of EPCR (sEPCR), resulting from the cleavage of the extracellular domain of the membrane bound EPCR (mEPCR) by a metalloprotease[18], can decrease the activation of PC by competing with mEPCR for PC[18]. sEPCR also inhibits APC anticoagulant activity by blocking the interaction with negatively charged membranes[19], an interaction that is necessary for effective inactivation of coagulation factors Va and VIIIa.

Currently the role of the EPCR/APC complex in renal transplantation is unknown; however APC has been extensively studied in inflammation settings and in sepsis. For example, Gupta *et al.* showed increased renal injury in rats with acquired PC deficiency in a polymicrobial sepsis model[20] and Keller *et al.* discovered that treatment with APC attenuates inflammation and preserves renal function during sepsis in rats[21].

There are two types of acute allograft rejection that can occur either separately or together: T-cell-mediated rejection (TCMR) and acute antibody-mediated rejection (ABMR). TCMR is the most common form of acute allograft rejection, caused by effector T-cells that infiltrate and proliferate in the graft (-draining lymph nodes) leading to graft rejection[22]. ABMR is caused by donor-specific antibodies and is characterised by histological changes such as leukocyte infiltration in the glomeruli and peritubular capillaries (PTC), tubular necrosis, congestion of PTC, infiltration of granulocytes, endothelial cell damage and finally fibrinoid arterial necrosis[23]. Damaged endothelial cells release injury molecules such as cytokines, chemokines, von Willebrand factor and P-selectin, which can induce leukocyte adhesion and activation of the complement and coagulation cascade[22]. As a result of activation of the complement cascade during ABMR currently one of the most reliable surrogate markers for ABMR is C4d positivity of PTC[23], which requires having access to biopsy material, involving an invasive procedure for the patient. In the clinical setting it is important to distinguish between patients with TCMR and ABMR, because the treatments of these two types of rejection are different[22].

In the current study we investigate the EPCR expression pattern in kidney transplants on both mRNA and protein level; and correlate plasma and urine sEPCR levels upon acute renal allograft rejection. We describe how urinary sEPCR can distinguish between ABMR and TCMR.

## Results

### Demographic and clinical characteristics of the patients

Table 1 shows the demographic and clinical characteristics of the patients. The three groups differed in some aspects. The patients in the ABMR group are slightly younger than those in the control group (p<0.05). As expected, the median serum creatinine concentration was higher and the estimated glomerular filtration rate (GFR) was lower in patients undergoing renal allograft rejection compared to patients without rejection (p<0.001).

**Table 1:** Clinical parameters of the included patients.

	Control Group (n = 33)	ABMR Group (n = 22)	TCMR Group (n = 26)
**Demographic characteristics:**			
Gender (M/F)	23/10	13/9	15/11
Age in years	49 (19–75) ^a^	40 (11–63) ^a^	48.5 (16–68)
Serum creatinine at the time of the biopsy ( µmol/L)	130 (69–334) §*	348 (84–881) §	335 (113–1341) *
Time (in days) between transplantation and renal biopsy	234 (0–771)	399.5 (5–6187)	47.5 (5–3675)
GFR (mL/min/1.73 m^2^)	48,78 (13,87–108.93) §*	13.93 (5.12–122.23) §	16.88 (3.48–61.28) *
Panel reactive antibody at the time of biopsy	0 (0–24) *	24 (0–100) *^a^	1 (0–78) ^a^
**Causes of primary kidney failure:**			
Polycystic kidney disease	7	7	3
Diabetes mellitus	0	1	0
Focal glomerulosclerosis	2	4	2
Hypertension	4	4	5
Immune complex mediated diseases	8	1	5
Vasculitis	1	0	1
Urinary tract infection	2	0	1
Other	9	5	9
**Donor characteristics:**			
Mean age in years (SD)	50,5 (16,2)	45.6 (16.6)	45.4 (11.9)
No. Cadaveric/No. Living	17/16	13/9	16/10
HLA mismatch	3 (0–6) §	4 (3–6) § ^a^	3 (0–6) ^a^

Date are shown as median and range unless stated otherwise

a: p<0.05 (Mann-Whitney Test)

§ , *: p<0.001 (Mann-Whitney Test)

**Figure 1:**
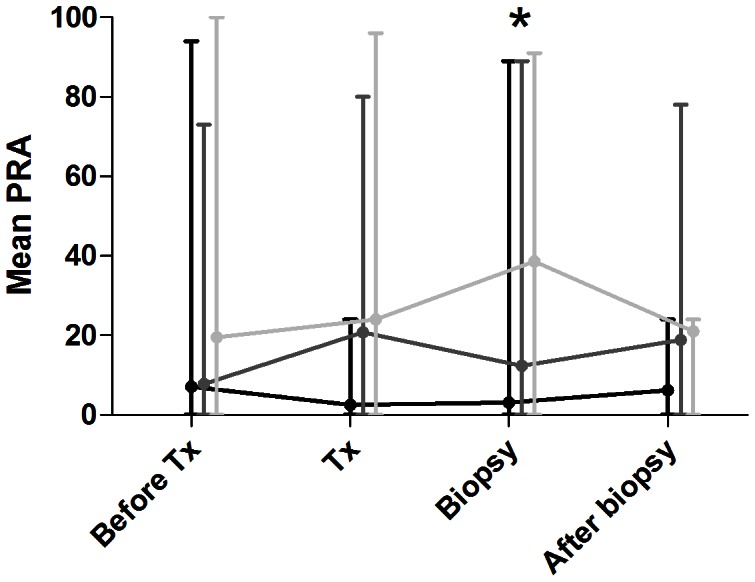
Evolution of the panel reactive antibody (PRA) levels in renal transplantation patients. PRA concentrations were measured before transplantation (before Tx), at the time of transplantation (Tx), at the time of biopsy and after the biopsy. PRA levels are shown for control (in black), antibody-mediated (ABMR, in light grey) and for T-cell-mediated rejection (TCMR, in dark grey) patients. PRA values within the groups did not differ significantly over time. However, at time of biopsy (i.e. during the acute rejection episode) PRA values in the ABMR group were significantly higher than in the TCMR group (P<0,05) and the control group (P<0,001). Results are shown as mean and range. * p<0.05 (Mann-Whitney Test)

More HLA mismatches were present in patients with ABMR compared with control (p<0.001) and TCMR groups (p<0.05) (Table 1). In our hospital, it was not standard care to evaluate donor-specific antibodies at time of rejection. However, panel reactive antibody (PRA) measurements were performed before transplantation, at the time of transplantation, at time of biopsy and after the biopsy. PRA levels did not change significantly over time within the 3 patient groups (multivariate analysis, figure 1). At time of biopsy however, the ABMR group showed significantly higher levels of PRA compared to control patients (p<0.001) and patients undergoing T-cell-mediated rejection (p<0.05, figure 1 and table 1).

All patients received immunosuppressive treatment consisting of CD25mAb (induction), corticosteroids, mycophenolate and a calcineurin inhibitor. Acute cellular rejections were treated with pulse doses of methylprednisolone 500 mg iv for 6 days. Antibody mediated rejections were treated with plasmapheresis for 7 days and rabbitATG (rATG). The starting dose of rATG was 5 mg/kg, 3 to 5 gifts were administered over 14 days. Dosages were titrated based on the total lymphocyte count after each administration (>300×10^9^/L: dose 5 mg/kg;>200×10^9^/L but <300×10^9^/L: dose 3 mg/kg; >150×10^9^/L but <200×10^9^/L: dose 2 mg/kg; <150×10^9^/L: no administration).

Table 2 shows the Banff scores of the patients. TCMR patients had more tubulitis and mononuclear cell interstitial inflammation than ABMR patients (p<0.05). 86% of the biopsies diagnosed as ABMR were C4d positive (p<0.001 versus TCMR and control).

**Table 2:** BANFF characteristics of the included patients.

	Control Group	ABMR Group	TCMR Group
Tubulitis	0 (0 – 1)* §	1 (0 – 3)* ^a^	1 (1 – 3)^a^ §
Mononuclear Cell Interstitial Inflammation	0 (0 – 1)* §	1 (0 – 3)* ^a^	2 (1 – 3)^a^ §
Glomerulitis	0 (0 – 2)* §	0,5 (0 – 3)*	1 (0 – 3)§
Arteriolar Hyaline Thickening	0 (0 – 2)	0 (0 – 2)	0 (0 – 2)
Intimal Arteritis	0 (0 – 0)* §	0 (0 – 3)*	0 (0 – 2)§
Glomerulopathy	0 (0 – 2)^a^	0 (0 – 3)^a^	0 (0 – 1)
Interstitial Fibrosis	0 (0 – 2)	0 (0 – 2)	0 (0 – 3)
Tubular Atrophy	1 (0 – 2)	1 (0 – 2)	1 (0 – 3)
Vascular Fibrous Intimal Thickening	0 (0 – 1)	1 (0 – 2)	0 (0 – 3)
Mesangial Matrix Increase	0 (0 – 1)	0 (0 – 3)	0 (0 – 3)
Capillaritis	0 (0 – 2)^a b^	0 (0 – 2)^a^	0 (0 – 2)^b^
No. C4d positive	0 (0%)*	19 (86%)* §	0 (0%)§

Data are shown as median and range.

a, b: p<0.05 (Mann-Whitney Test)

* , §: p<0.001 (Mann-Whitney Test)

### EPCR mRNA levels in kidney transplant biopsies

**Figure 2:**
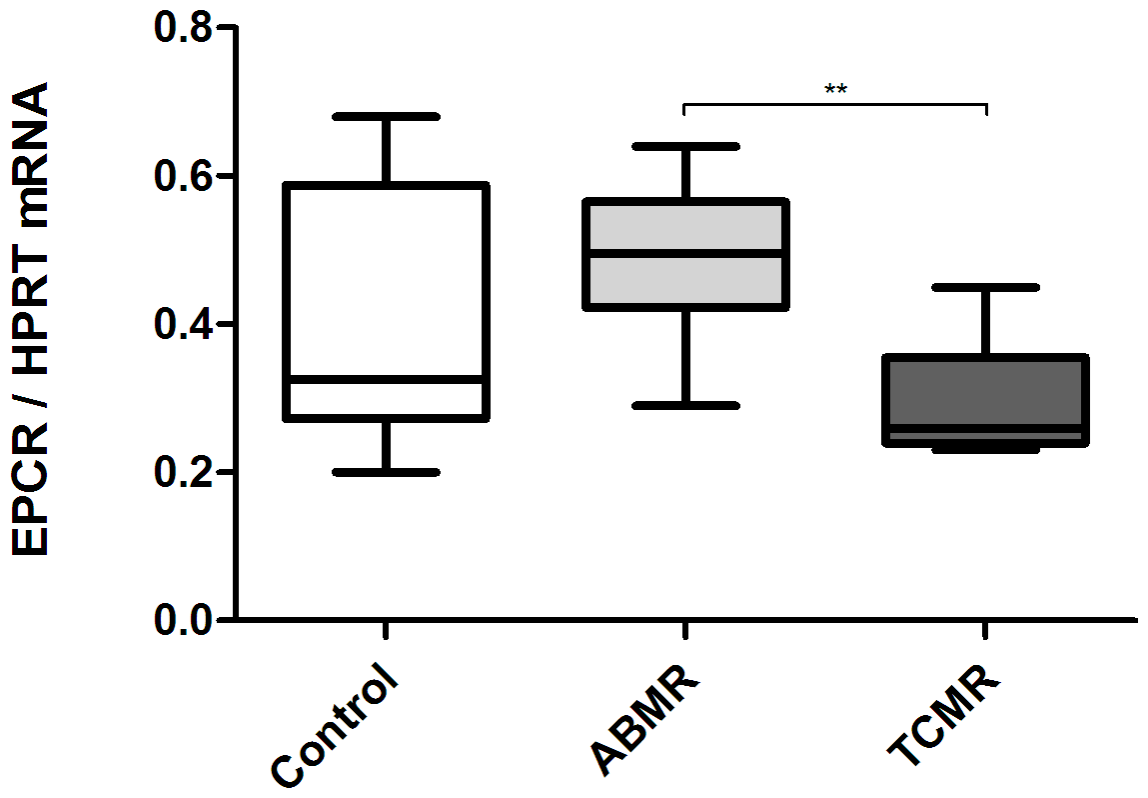
EPCR mRNA expression in kidney transplant. EPCR mRNA levels were measured with qPCR on whole kidney biopsies. Results are shown as ratio between EPCR and HPRT Ct. Antibody-mediated (ABMR) rejection patients showed higher levels of EPCR mRNA than T-cell-mediated rejection (TCMR) patients. Results are shown as median, interquartile range and range. ** p<0.001 (Mann-Whitney Test)

As shown in figure 2, PROCR/HPRT1 (EPCR) mRNA ratios were significantly higher in patients with ABMR (0.49 [0.29 – 0.64]) compared to those in patients with TCMR (0.26 [0.23 – 0.45], p<0.001) but not compared with control patients (0.33 [0.20 – 0.68], p>0.05).

### EPCR protein expression in kidney transplants

**Figure 3:**
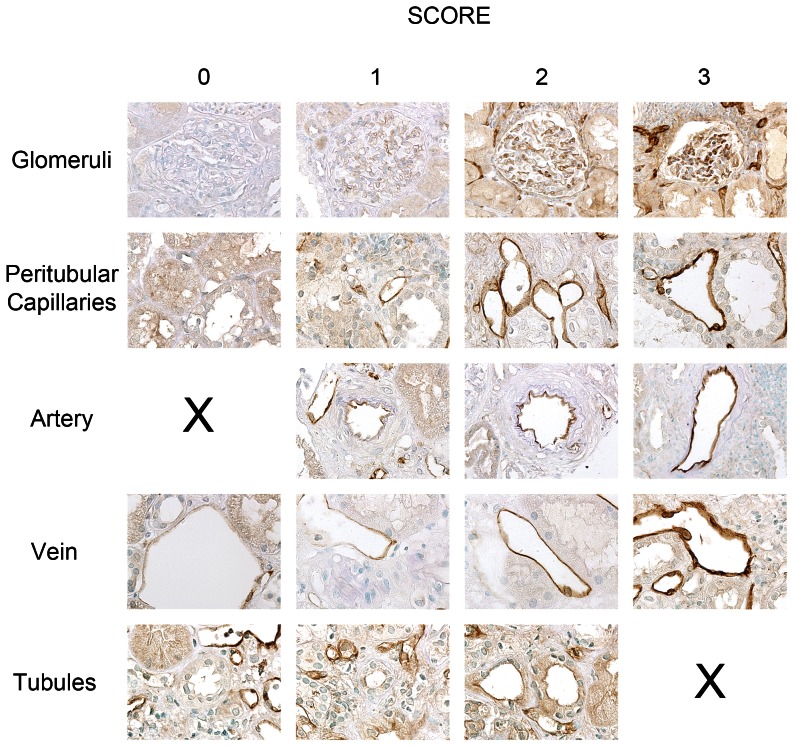
EPCR expression patterns in transplant biopsies. Representative immunostainings of kidney biopsies for EPCR in glomeruli (×32 magnification), peritubular capillaries (×64), arteries (×64), veins (×64) and tubules (x64). Arteries were always positive; therefore no picture with a score of 0 is shown. For tubules no score of 3 was assigned.

In order to visualize the expression pattern of EPCR in transplant biopsies, we performed immunostainings. Figure 3 shows representative EPCR staining patterns in kidney transplant biopsies. Intensity of staining was evaluated in five kidney substructures: glomeruli, peritubular capillaries, arteries, veins and tubules on a semi quantitative scale from 0 to 3[24]. In general, arteries were more intensely stained than other substructures. Therefore no score of 0 was assigned for arteries. Intensity of staining was generally weaker in tubules than in other kidney compartments, no staining intensity corresponding to a score of 3 was assigned for tubules.

**Figure 4:**
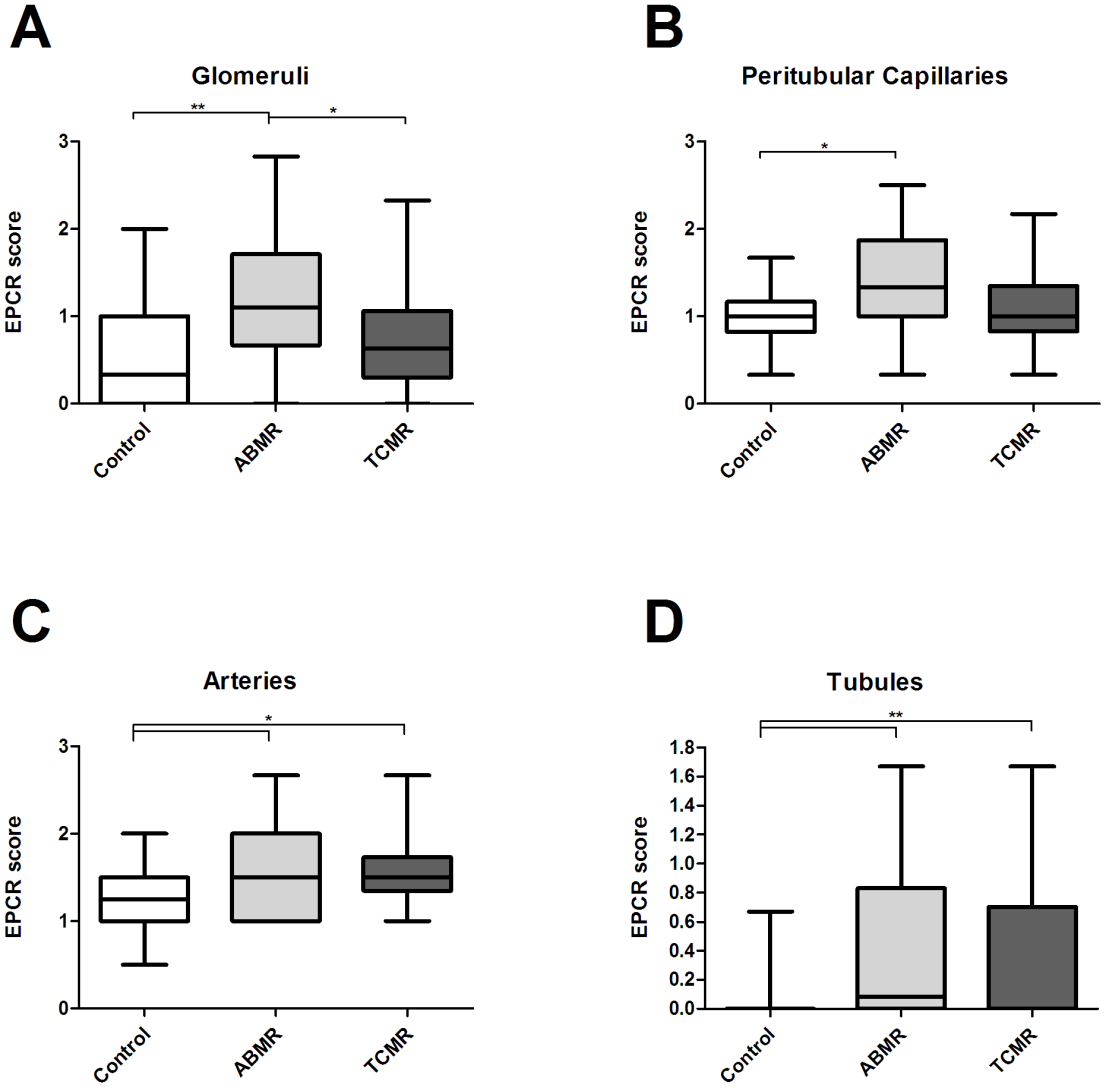
EPCR levels of expression in kidney transplant biopsies. Semi quantitative scores (on a scale from 0 to 5) of EPCR immunostainings. EPCR expression on glomeruli (A) and peritubular capillaries (B) are significantly elevated in ABMR patients than in TCMR or control patients. EPCR expression is higher in arteries (C) and in tubules (D) of rejection patients than in control patients. Results are shown as median, interquartile range and range. * p<0.05 (Mann-Whitney Test) ** p<0.001 (Mann-Whitney Test)

EPCR expression was significantly higher in patients with ABMR compared to patients without rejection or with TCMR in glomeruli (p<0.001 and p<0.05, respectively, figure 4A). In capillaries ABMR patients showed higher EPCR expression only compared to control (p<0.05, figure 4B).

In arteries we observed higher EPCR expression in patients with ABMR and TCMR compared to patients without rejection (p<0.05, figure 4C). The same expression pattern was observed in tubules (p<0.05, figure 4D)

EPCR scores of the venous endothelium did not show any differences between ABMR, TCMR and control (data not shown).

### Plasmatic and urinary sEPCR concentration

Plasma and urine levels of sEPCR were determined by ELISA. The urinary concentration of sEPCR was corrected for dilution. We confirmed the presence of intact sEPCR protein, and not degradation products by western blot analysis (data not shown).

**Figure 5:**
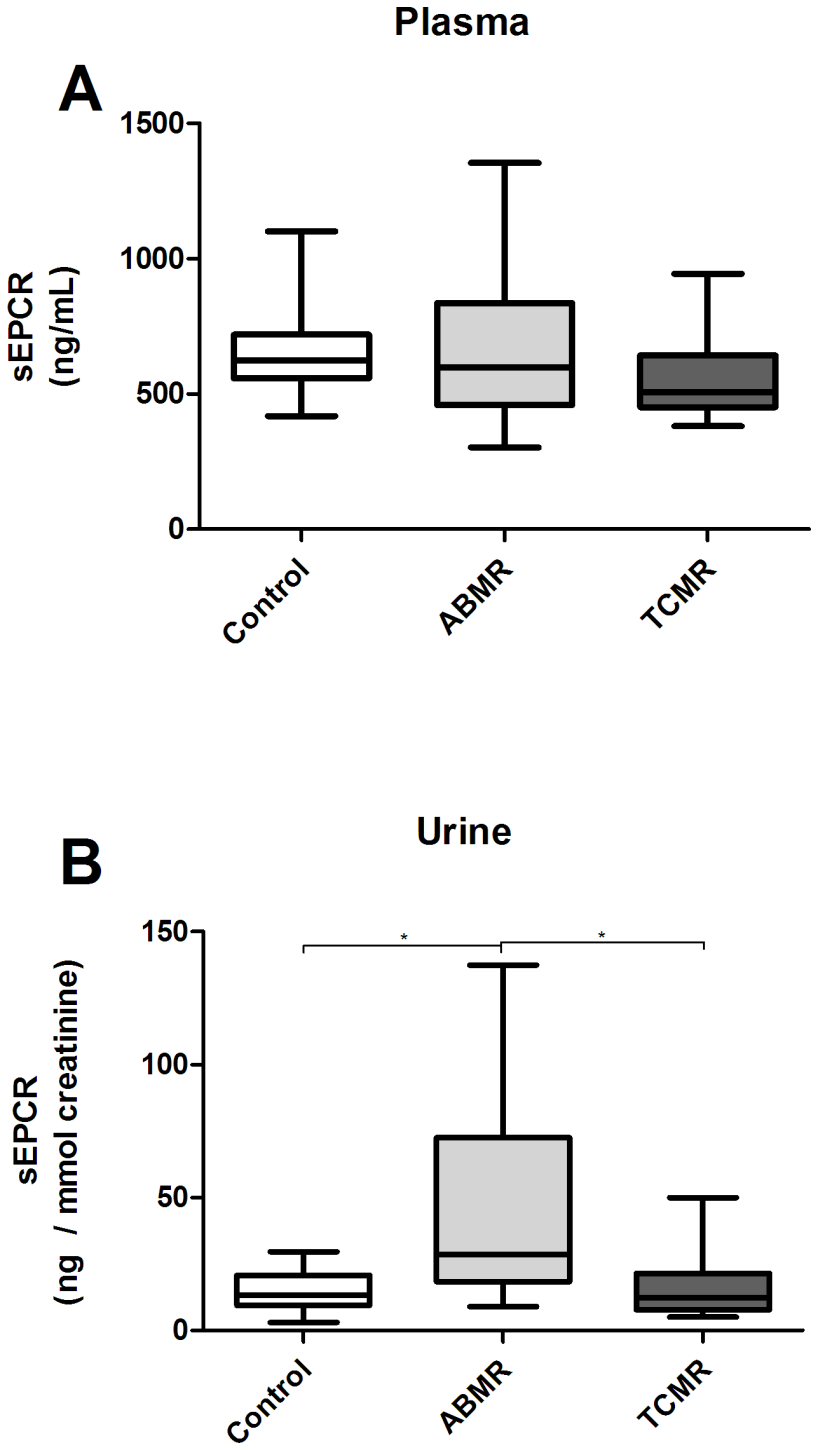
sEPCR in serum (A) and urine (B) of transplant patients. Plasma and urine sEPCR concentration were measured with ELISA, urinary sEPCR concentration is corrected for the urine dilution by dividing the concentration by the urinary creatinine concentration. Plasma sEPCR levels did not differ. Urine sEPCR levels are elevated in the ABMR group than in patients with TCMR or without rejection. Results are shown as median, interquartile range and range. * p<0.01 (Mann-Whitney Test)

Plasma sEPCR levels were not significantly different between ABMR patients (599 ng/mL [67 - 1355]), control patients (623 ng/mL [418 – 1102], p>0.05) or patients with TCMR (508 ng/mL [381 – 945], p>0.05) (Figure 5A). Conversely, the urine levels of sEPCR were significantly higher in patients with ABMR (29 ng/mmol creatinine [9 – 137]) than in either patients with TCMR (12 ng/mmol creatinine [5 – 50], p<0.05) or without rejection (13 ng/mmol creatinine [3]–[30], p<0.01, Figure 5B). The urine creatinine concentration did not differ between the groups (p = 0.6).

We found no correlation between protein EPCR scores in the kidney and sEPCR concentration in plasma or urine (data not shown).

### Association of clinical parameters and Banff scores with EPCR

**Figure 6:**
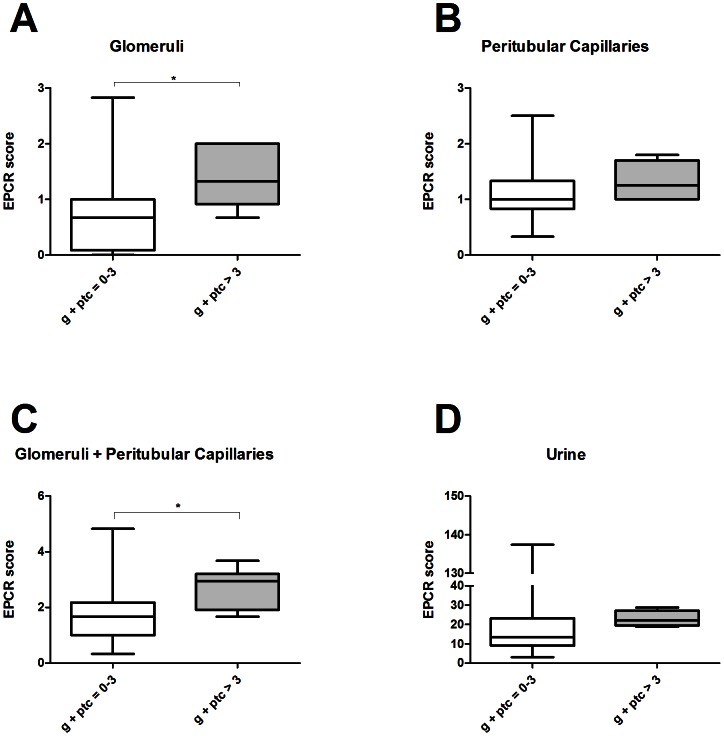
Association between microcirculatory inflammation and EPCR. Severe antibody-mediated inflammation of the microcirculation of the transplant, defined as the composite of Banff g and ptc scores [Sis et al. 2012 Am J Transplant], associated with higher EPCR scores in both the glomeruli (A) and the peritubular capillaries (B) as well as the composite EPCR score of glomeruli and peritubular capillaries (C). Severe microcirculatory inflammation associated with higher levels of soluble EPCR in the urine at time of biopsy. Data are represented as box-and-whisker plots with median and (interquartile) range. * p<0.05 (Mann-Whitney Test)

Recently, Sis et al.[25] proposed that the cumulative Banff score for glomerulitis and peritubular capillaritis (g+ptc) associates with antibody-mediated inflammation of the microcirculatory circuit, irrespective of C4d positivity. Indeed, C4d-negative ABMR is an increasingly recognised entity. We investigated whether little (g+ptc≤3) or severe (g+ptc>3) microcirculatory inflammation related to higher levels of urine sEPCR or staining intensity of EPCR in these renal structures. Glomerular EPCR (p = 0.009) and the composite of glomerular and peritubular capillary EPCR scores (p = 0.009) significantly associated with severe microcirculatory inflammation (g+ptc>3). Higher scores for EPCR on the peritubular capillaries tended to relate to higher microcirculatory inflammation as well (p = 0.09). Not for each biopsy sample, a matching urine sample was available. Therefore we imputed the missing values with multivariate bootstrap methods. Complete case analysis showed a trend for higher urine sEPCR levels at the time of biopsy when severe microcirculatory inflammation was present (p = 0.06) (figure 6).

### Receiver Operating Characteristic (ROC) analysis

In order to evaluate the possible usefulness of urinary sEPCR as a non-invasive biomarker for ABMR, a ROC calculation was performed. The area under the ROC curve was used to summarize the discriminative ability of the test, the closer to 1 the better.

**Figure 7:**
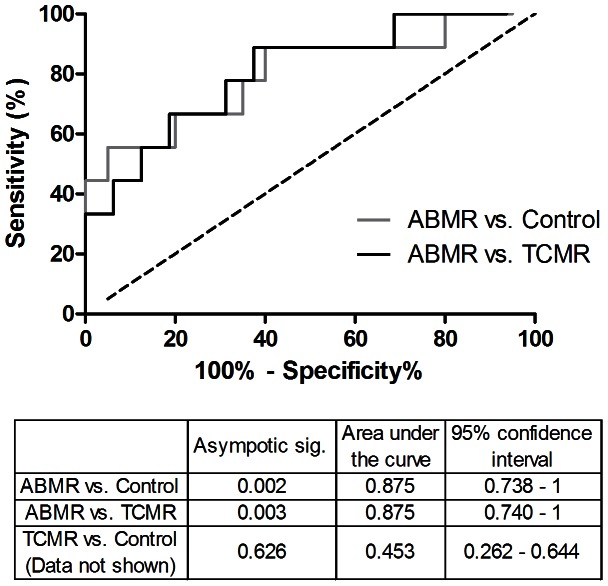
ROC analyses, graphical representation. Receiver operating characteristic (ROC) analysis of urine sEPCR concentration for the prediction of ABMR in kidney transplantation. ROC analysis demonstrated that urinary sEPCR is appropriate to discriminate between ABMR patients and TCMR or control patients. In grey: ABMR vs. Control, in black: ABMR vs. TCMR.

As shown in figure 7, the sEPCR concentration in urine can be used to discriminate ABMR patients from TCMR patients (area under the curve of 0.875, p<0.01) and patients without rejection (area under the curve of 0.8785, p<0.01).

The cut-off value with the highest combined sensitivity and specificity for discriminating between ABMR patients and control patients was at 21.6 ng/mmol creatinine (75% sensitivity, 80% specificity). The optimum sEPCR concentration for discriminating between ABMR and TCMR patients was 22.1 ng/mmol creatinine (75% sensitivity, 82% specificity). The dotted line represents the line of equal sensitivity and specificity.

## Discussion

This study is the first to describe the levels of membrane bound and soluble EPCR during acute renal allograft rejection in a transplant patient cohort.

We observed higher EPCR mRNA levels in ABMR patients compared with TCMR patients. On the protein level, we observed in glomeruli more EPCR expression in ABMR patients compared with patients without rejection or with TCMR. In the peritubular capillaries EPCR expression was higher in ABMR patients than in control patients. Immunostaining also revealed a higher expression of EPCR in arteries and tubules from patients with ABMR and TCMR compared with patients without rejection. Finally we found increased concentrations of sEPCR in the urine of ABMR patients compared with patients with TCMR or without rejection. Although validation is needed, these findings together with the ROC analysis indicate that urinary sEPCR could be used as a non-invasive biomarker for antibody mediated kidney rejection.

There are some limitations to our study. Since it is a retrospective study some patients could not be studied for all the different tests. Furthermore the ABMR group contains a relatively small number of patients with urine sample available. The reason for this is the low incidence of ABMR within the renal transplant patient population in our institution. Nevertheless even with a population of 8 samples from the ABMR group and 21 from the TCMR group the ROC analysis achieves 80% power to detect a difference of 0.422 between the area under the curve under the null hypothesis of 0.5000 and an AUC under the alternative hypothesis of 0.875 using a two-sided z-test at a significance level of 0.05.

Another limitation of our study may be the lack of data on donor specific antibodies (DSA). ABMR diagnosis can be complex in the clinical setting, often requiring DSA. Indeed, C4d-negative ABMR is an increasingly recognized phenomenon[25]. DSA are not routinely determined in our institute. Instead, we have incorporated the less specific PRA data in our study, showing that ABMR patients have higher percentages of PRA than TCMR patients at time of biopsy, i.e. at time of the acute rejection episode.

It is known from the literature that the expression of EPCR is not only limited on endothelial cells. On the mRNA level EPCR is transcribed in HUVEC[26], in a human alveolar epithelium cell line[27], in rat lung[28], pancreatic tissue[29], in gastric epithelial cells[30] and in immortalized human proximal tubular epithelial cell line[31]. In this study we show for the first time EPCR mRNA in kidney allograft tissue. On the protein level EPCR is known to be expressed on HUVEC[26], endothelium in the heart, the lung, the kidney, the skin and on other organs[4] but also on epithelial cells such as a prostatic cancer cell line[32], human alveolar epithelium[27], rat lung[28], pancreatic tissue[29] and gastric epithelial cells[30]. In normal renal tissue, EPCR is expressed on vein and arterial endothelium[4]. Although the expression of EPCR by tubular epithelial cells has been described in a cell line of artificially immortalized kidney cells[31], our study is the first to actually demonstrate EPCR expression by tubular epithelial cells in human tissue.

The roles of EPCR and protein C have been extensively studied in sepsis setting. EPCR and APC form a complex with PAR-1 and initiate biological effects such as anticoagulant, anti-inflammatory, anti-apoptotic and cytoprotective activities in vitro and in vivo[3], [8], [9], [10], [11], [12], [13], [14], [15], [16], [17]. The APC/EPCR/PAR-1 axis inhibits the release of inflammatory mediators[9], [10], [11], [12], down-regulates the expression of adhesion molecules[13], inhibits neutrophil and eosinophil migration[3], [14] and exerts protective activities against infection. APC-EPCR-PAR1 interaction has been shown to play a protective role in several conditions such as in a sepsis model using *E.coli* [33] and was shown to have anti-apoptotic activities[13], [15] and protect endothelial barrier function[16], [17].

Importantly, Song *et al.* showed an upregulation of EPCR expression in the kidney of mice injected with LPS[34].

These findings clearly indicate that EPCR is part of a protective pathway. Considering this we assume that the higher EPCR expression during acute kidney rejection could be part of a protective mechanism for the graft. EPCR could exert its protective activities on several levels.

Firstly, higher EPCR expression could contribute to enhanced APC formation and therefore more activation of PAR-1 and its associated protective properties. This could suggest that an actively regulated protection mechanism involving the EPCR/APC/PAR-1 signaling cascade is taking place in the setting of acute allograft rejection.

Secondly, it is known that ABMR is characterized by activation of the coagulation cascade, resulting in elevated levels of FVIIa[22] which can bind to EPCR and activates its associated protective activities[35].

EPCR not only exists as a membrane bound receptor but also as a soluble protein. sEPCR has a comparable affinity for APC as mEPCR[19]. sEPCR can compete with mEPCR for APC, resulting in a decreased activation of PC[18]. sEPCR also inhibits APC anticoagulant activity by blocking the interaction with negatively charged membranes[19], a necessary interaction for an effective inactivation of coagulation factors Va and VIIIa. Currently, very little is known about sEPCR in transplantation. The only study on sEPCR in transplantation was performed by Keven *et al*, who measured plasma sEPCR concentration before and after kidney transplantation in patients without rejection[36]. Three months after transplantation the serum sEPCR concentration was significantly lower than before the transplant procedure. From this, Keven *et al.* concluded that the elevated pre-transplant sEPCR levels reflected endothelial damage, due to the hemo-dialysis regimen before transplantation[36]. Elevated levels of sEPCR have been described in vasculitis and SLE, which has fostered the hypothesis that sEPCR may be a biomarker for endothelial dysfunction[37].

Considering this, it is not surprising to observe higher sEPCR production during rejection, knowing that allograft rejection is associated with endothelial damage and dysfunction especially in antibody mediated rejection[22]. Until now sEPCR concentration was only measured in plasma, in this study we are the first to show sEPCR presence in the urine of patients. The origin of the urinary sEPCR is unclear. The relatively low molecular weight of sEPCR (about 43 kDA[38]) makes its filtration through the glomeruli theoretically possible. Therefore sEPCR could be derived either from filtrated blood or from shed tubular mEPCR. Shedding of monocytes or neutrophils bound EPCR might also be an alternative source of urinary sEPCR.

Renal transplantation is the most suitable therapy for end stage kidney disease. Despite recent progress in anti-rejection therapy, approximately 23% of transplanted patients undergo an episode of acute rejection within the first year post-transplant[39]. For all patients undergoing a rejection episode it is crucial to have the ability to correctly diagnose the presence of rejection. Therefore having access to a convenient biomarker could be of interest for the clinical practice. Currently, sampling of a kidney biopsy and its histological examination is the gold standard for diagnosing both TCMR and ABMR, causing discomfort for the patient.

Therefore we propose that urinary sEPCR could be of interest for diagnosing acute kidney antibody mediated rejection as proven by the ROC analysis which demonstrates that urinary sEPCR might be suitable to make the distinction between ABMR patients and non-rejecting patients (AUC = 0.875, p = 0.002) and importantly also between ABMR and TCMR patients (AUC = 0.875, p = 0.003). Although validation will be needed in a larger patient cohort, we conclude that urinary sEPCR could be a suitable candidate to diagnose antibody mediated renal rejection in the clinical setting, in a non-invasive way.

## Methods

### Patients

Eighty-one patients who underwent kidney transplantation between 1994 and 2008 were retrospectively selected from the patient population of the Academic Medical Center at the University of Amsterdam. Patients were selected based on pathological diagnosis. Renal biopsies that fulfilled the minimal criteria for diagnostic assessment (7 glomeruli and at least 1 artery) according to the Banff 1997 criteria were available from all patients[40]. All biopsies were stained for Haematoxylin-Eosin, Periodic Acid Schiff Diastase and Methenamine Silver (Jones) and scored following the Banff 2007 guidelines[41].

Patients were divided in 3 groups, according to the biopsy diagnosis. The TCMR group consisted of 26 patients with interstitial infiltration, tubulitis or intimal arteritis [40]. Twenty patients with acute-tubulus-necrosis-like inflammation, capillary and/or inflammation and/or thrombosis, arteritis and C4d tubular deposition were assigned to the ABMR group[40]. In addition, two patients with signs of ABMR-associated microvascular inflammation[25] with negative C4d staining were assigned to the ABMR group. Finally, the control group consisted of 33 patients without signs of rejection. Kidney material of the control group came from protocol biopsies and showed no signs of inflammation or rejection.

Time matched mRNA, derived from frozen transplant biopsies, was available for 14 patients in the control group, 13 in the TCMR group and 11 in the ABMR group as well as matched serum samples for 21 patients in the control group, 22 in the TCMR group and 9 in the ABMR group. Matched urine samples (taken from 24 hours urine samples) were available for 22 patients in the control group, 21 in the TCMR group and 8 in the ABMR group.

Circulating donor specific antibodies are not systematically measured in kidney transplant patients in our institution. Consequently, according to the Banff criteria[41], we considered a patient belonging to the ABMR group when showing a diffuse C4d staining of the peritubular capillaries on frozen section. If frozen sections were not available, we performed C4d staining on paraffin sections, patients with more than 50% of positive peritubular capillaries were considered as positive for ABMR.

Seven patients who showed signs of both TCMR and ABMR were included in the ABMR group.

All biological material was collected for previous studies[42], [43] for which written informed consent was given by all patients for their information to be stored in the hospital database and to be used for research. This research project used left-over biological material, anonymised and delinked from patient records, and as such was not subject to any requirement for ethical review or approval.

### RNA Extraction and Processing for Real-Time PCR

mRNA from complete kidney tissue was extracted from frozen renal biopsies cut into 25 µm thick sections using a Microm HM500 cryostat (Adamas Instruments BV) and collected in an Eppendorf tube containing TRIzol (Invitrogen, Breda, The Netherlands). After 5 minutes incubation at room temperature RNA was extracted using chloroform. cDNA was synthesised using a standard procedure.

Real-time reverse-transcriptase polymerase chain reaction (RT-PCR) was performed on a Lightcycler® 480 Real-Time PCR System using Lightcycler® 480 SYBR Green I Master (Roche Applied Science, Mijdrecht, the Netherlands). Specific primers were designed (synthesized by Eurogentec, Liège, Belgium) for human PROCR (EPCR) (forward ACCTTGGCCTTTCCTCTGAC, reverse CTCCCATTCACAGCCACTTC). Results were analyzed using LinRegPCR 12.4 software (Heart Failure Research Center, Academic Medical Center, Amsterdam, the Netherlands). PROCR gene expression was normalized against two different housekeeping genes: HPRT1 (hypoxanthine phosphoribosytransferase 1) and ACTBL2 (β-actin). Comparable results were obtained with the two housekeeping genes. We decided to present the ratios between Ct values of PROCR and HPRT1 (forward TTGTTGGATATGCCCTTGACT, reverse CCGCTGTCTTTTAGGCTTTG).

### Immunostaining

C4d staining was performed on frozen sections if available using a mouse anti-human C4d antibody (AbD Serotec, Dusseldorf, Germany, ref. 2222-8004). Alternatively, C4d staining was performed on paraffin sections using a rabbit anti-human C4d antibody (Cell Marque, Rocklin, USA, ref. 404A-14), as described previously[43].

Paraffin sections of kidney biopsies were immunostained for EPCR. Antigen retrieval with Tris EDTA pH 9 (20 minutes at 121°C) was performed for optimal staining. 4 µm thick sections were incubated for 60 hours at 4°C with goat anti human EPCR monoclonal antibody (452 ng/mL, kind gift of Dr. C. Esmon, Oklahoma Medical Research Foundation). Primary antibody binding was detected with a peroxydase kit (30 minutes incubation at room temperature, Powervisoin poly HRP-Anti-mouse IgG, Immunologic, Duiven, Netherlands). Staining was developed with Ultra DAB (Immunologic, Duiven, Netherlands).

On EPCR stained sections, the intensity of immunostaining was scored semiquantitatively on a scale from 0–3 (respectively absent, weak, moderate or strong) following the method of Faust *et al.* [24]. Intensity of staining was evaluated in five kidney substructures: glomeruli, peritubular capillaries, arteries, veins and tubules. All sections were coded and scored by two blinded investigators. For each section a mean score was calculated from at least three high power fields. C4d staining was evaluated following the Banff 2007 recommendations[41] by a blinded pathologist.

### Enzyme-linked immunosorbent assay (ELISA)

Twenty µL aliquots of urine and plasma were pre-treated with 10 µL 1N HCl. After 10 minutes of incubation at room temperature acidified samples were neutralized with 9 µL 1N NaOH. Prior to the assay samples were diluted with Calibrator Diluent (RD5–24 provided with the ELISA kit) to reach a total dilution factor of 15.6 for urine samples and 39 for plasma samples. Soluble EPCR concentrations in urine and plasma were measured using Human EPCR Quantikine kit (R&D System, Abingdon, UK) according to the manufacturer's protocol. According to the data sheet the mean minimal detectable doses is 0.064 ng/mL. Optical densities were measured using a microplate reader set to 450 nm and were corrected with a wavelength of 570 nm. A standard curve was created using the trial version of MasterPlex® ReaderFit software (Hitachi Solution America Ltd.), capable of generating a four parameter logistic curve fit.

The urinary concentration of sEPCR was corrected for the urine dilution by dividing the urinary concentration of sEPCR by the urinary creatinine concentration (urine creatinine concentration did not differ between the groups, data not shown).

### Glomerular Filtration Rate (GFR)

The GFR was estimated with use of the CKD-EPI formula [44].

### Statistical Analysis

All data sets were tested for their distribution prior to analyses.

Data are expressed as median and range unless stated otherwise. Wilcoxon-Mann Whitney test, Kruskal Wallis, Spearman's correlation test, the multivariate analysis and Receiver Operating Characteristic (ROC) analyse were performed using SPSS 19 software (IBM Corporation, Stomer NY USA) and the R computing environment (www.r-project.org). Overall a two-tailed p-value of <0.05 was considered significant.

## References

[1] Fukudome K, Esmon CT (1995) Molecular cloning and expression of murine and bovine endothelial cell protein C/activated protein C receptor (EPCR). The structural and functional conservation in human, bovine, and murine EPCR. J Biol Chem 270: 5571–5577. 7890676 10.1074/jbc.270.10.5571

[2] Galligan L, Livingstone W, Volkov Y, Hokamp K, Murphy C, et al (2001) Characterization of protein C receptor expression in monocytes. Br J Haematol 115: 408–414. 11703343 10.1046/j.1365-2141.2001.03187.x

[3] Sturn DH, Kaneider NC, Feistritzer C, Djanani A, Fukudome K, et al (2003) Expression and function of the endothelial protein C receptor in human neutrophils. Blood 102: 1499–1505. 12714492 10.1182/blood-2002-12-3880

[4] Laszik Z, Mitro A, Taylor FB Jr, Ferrell G, Esmon CT (1997) Human protein C receptor is present primarily on endothelium of large blood vessels: implications for the control of the protein C pathway. Circulation 96: 3633–3640. 9396465 10.1161/01.cir.96.10.3633

[5] Regan LM, Mollica JS, Rezaie AR, Esmon CT (1997) The interaction between the endothelial cell protein C receptor and protein C is dictated by the gamma-carboxyglutamic acid domain of protein C. J Biol Chem. 272: 26279–26284. 10.1074/jbc.272.42.26279 9334197

[6] Taylor FB Jr, Peer GT, Lockhart MS, Ferrell G, Esmon CT (2001) Endothelial cell protein C receptor plays an important role in protein C activation in vivo. Blood 97: 1685–1688. 11238108 10.1182/blood.v97.6.1685

[7] Walker FJ, Fay PJ (1992) Regulation of blood coagulation by the protein C system. FASEB J 6: 2561–2567. 1317308 10.1096/fasebj.6.8.1317308

[8] Riewald M, Petrovan RJ, Donner A, Mueller BM, Ruf W (2002) Activation of endothelial cell protease activated receptor 1 by the protein C pathway. Science 296: 1880–1882. 12052963 10.1126/science.1071699

[9] White B, Schmidt M, Murphy C, Livingstone W, O'Toole D, et al (2000) Activated protein C inhibits lipopolysaccharide-induced nuclear translocation of nuclear factor kappaB (NF-kappaB) and tumour necrosis factor alpha (TNF-alpha) production in the THP-1 monocytic cell line. Br J Haematol 110: 130–134. 10930989 10.1046/j.1365-2141.2000.02128.x

[10] Yuksel M, Okajima K, Uchiba M, Horiuchi S, Okabe H (2002) Activated protein C inhibits lipopolysaccharide-induced tumor necrosis factor-alpha production by inhibiting activation of both nuclear factor-kappa B and activator protein-1 in human monocytes. Thromb Haemost 88: 267–273. 12195699

[11] Brueckmann M, Hoffmann U, De Rossi L, Weiler HM, Liebe V, et al (2004) Activated protein C inhibits the release of macrophage inflammatory protein-1-alpha from THP-1 cells and from human monocytes. Cytokine 26: 106–113. 15135804 10.1016/j.cyto.2004.01.004

[12] Grey ST, Tsuchida A, Hau H, Orthner CL, Salem HH, et al (1994) Selective inhibitory effects of the anticoagulant activated protein C on the responses of human mononuclear phagocytes to LPS, IFN-gamma, or phorbol ester. J Immunol 153: 3664–3672. 7523500

[13] Joyce DE, Gelbert L, Ciaccia A, DeHoff B, Grinnell BW (2001) Gene expression profile of antithrombotic protein c defines new mechanisms modulating inflammation and apoptosis. J Biol Chem 276: 11199–11203. 11278252 10.1074/jbc.C100017200

[14] Feistritzer C, Sturn DH, Kaneider NC, Djanani A, Wiedermann CJ (2003) Endothelial protein C receptor-dependent inhibition of human eosinophil chemotaxis by protein C. J Allergy Clin Immunol. 112: 375–381. 10.1067/mai.2003.1609 12897745

[15] Mosnier LO, Griffin JH (2003) Inhibition of staurosporine-induced apoptosis of endothelial cells by activated protein C requires protease-activated receptor-1 and endothelial cell protein C receptor. Biochem J 373: 65–70. 12683950 10.1042/BJ20030341 PMC1223481

[16] Feistritzer C, Riewald M (2005) Endothelial barrier protection by activated protein C through PAR1-dependent sphingosine 1-phosphate receptor-1 crossactivation. Blood 105: 3178–3184. 15626732 10.1182/blood-2004-10-3985

[17] Finigan JH, Dudek SM, Singleton PA, Chiang ET, Jacobson JR, et al (2005) Activated protein C mediates novel lung endothelial barrier enhancement: role of sphingosine 1-phosphate receptor transactivation. J Biol Chem 280: 17286–17293. 15710622 10.1074/jbc.M412427200

[18] Xu J, Qu D, Esmon NL, Esmon CT (2000) Metalloproteolytic release of endothelial cell protein C receptor. J Biol Chem 275: 6038–6044. 10681599 10.1074/jbc.275.8.6038

[19] Liaw PC, Neuenschwander PF, Smirnov MD, Esmon CT (2000) Mechanisms by which soluble endothelial cell protein C receptor modulates protein C and activated protein C function. J Biol Chem 275: 5447–5452. 10681521 10.1074/jbc.275.8.5447

[20] Gupta A, Berg DT, Gerlitz B, Sharma GR, Syed S, et al (2007) Role of protein C in renal dysfunction after polymicrobial sepsis. J Am Soc Nephrol 18: 860–867. 17301189 10.1681/ASN.2006101167

[21] Keller SA, Moore CC, Evans SL, McKillop IH, Huynh T (2011) Activated protein C alters inflammation and protects renal function in sepsis. J Surg Res 168: e103–109. 21429520 10.1016/j.jss.2011.01.008

[22] Nankivell BJ, Alexander SI (2010) Rejection of the kidney allograft. N Engl J Med 363: 1451–1462. 20925547 10.1056/NEJMra0902927

[23] Colvin RB (2007) Antibody-mediated renal allograft rejection: diagnosis and pathogenesis. J Am Soc Nephrol 18: 1046–1056. 17360947 10.1681/ASN.2007010073

[24] Faust SN, Levin M, Harrison OB, Goldin RD, Lockhart MS, et al (2001) Dysfunction of endothelial protein C activation in severe meningococcal sepsis. N Engl J Med 345: 408–416. 11496851 10.1056/NEJM200108093450603

[25] Sis B, Jhangri GS, Riopel J, Chang J, de Freitas DG, et al (2012) A new diagnostic algorithm for antibody-mediated microcirculation inflammation in kidney transplants. Am J Transplant 12: 1168–1179. 22300601 10.1111/j.1600-6143.2011.03931.x

[26] Gao XH, Xu XX, Pan R, Li Y, Luo YB, et al (2009) Saponin fraction from Astragalus membranaceus roots protects mice against polymicrobial sepsis induced by cecal ligation and puncture by inhibiting inflammation and upregulating protein C pathway. J Nat Med 63: 421–429. 19548065 10.1007/s11418-009-0348-2

[27] Wang L, Bastarache JA, Wickersham N, Fang X, Matthay MA, et al (2007) Novel role of the human alveolar epithelium in regulating intra-alveolar coagulation. Am J Respir Cell Mol Biol 36: 497–503. 17099142 10.1165/rcmb.2005-0425OC PMC1899324

[28] Lu ZQ, He XY, Hong GL, He F, Liang H, et al (2009) [Expression of thrombomodulin, endothelial protein C receptor in lung tissue of acute paraquat poisoned rats and intervention of sodium dimercaptopropane sulfonate]. Zhonghua Lao Dong Wei Sheng Zhi Ye Bing Za Zhi 27: 453–456. 20095324

[29] Ping C, Yongping Z, Minmin Q, Weiyan Y, Yaozong Y (2010) Activated protein C improves the severity of severe acute pancreatitis via up-regulating the expressions of endothelial cell protein C receptor and thrombomodulin. Dig Dis Sci 55: 1599–1609. 19680809 10.1007/s10620-009-0909-y

[30] Nakamura M, Gabazza EC, Imoto I, Yano Y, Taguchi O, et al (2005) Anti-inflammatory effect of activated protein C in gastric epithelial cells. J Thromb Haemost 3: 2721–2729. 16246255 10.1111/j.1538-7836.2005.01635.x

[31] Bae JS, Kim IS, Rezaie AR (2010) Thrombin down-regulates the TGF-beta-mediated synthesis of collagen and fibronectin by human proximal tubule epithelial cells through the EPCR-dependent activation of PAR-1. J Cell Physiol 225: 233–239. 20506163 10.1002/jcp.22249 PMC3957190

[32] Menschikowski M, Hagelgans A, Tiebel O, Klinsmann L, Eisenhofer G, et al (2011) Expression and shedding of endothelial protein C receptor in prostate cancer cells. Cancer Cell Int 11: 4. 21320357 10.1186/1475-2867-11-4 PMC3045874

[33] Taylor FB Jr, Stearns-Kurosawa DJ, Kurosawa S, Ferrell G, Chang AC, et al (2000) The endothelial cell protein C receptor aids in host defense against Escherichia coli sepsis. Blood 95: 1680–1686. 10688824

[34] Song D, Ye X, Xu H, Liu SF (2009) Activation of endothelial intrinsic NF-{kappa}B pathway impairs protein C anticoagulation mechanism and promotes coagulation in endotoxemic mice. Blood 114: 2521–2529. 19620400 10.1182/blood-2009-02-205914 PMC2746475

[35] Sen P, Gopalakrishnan R, Kothari H, Keshava S, Clark CA, et al (2011) Factor VIIa bound to endothelial cell protein C receptor activates protease activated receptor-1 and mediates cell signaling and barrier protection. Blood 117: 3199–3208. 21252088 10.1182/blood-2010-09-310706 PMC3062318

[36] Keven K, Elmaci S, Sengul S, Akar N, Egin Y, et al. (2009) Soluble endothelial cell protein C receptor and thrombomodulin levels after renal transplantation. Int Urol Nephrol. 10.1007/s11255-009-9654-6 19802701

[37] Sesin CA, Yin X, Esmon CT, Buyon JP, Clancy RM (2005) Shedding of endothelial protein C receptor contributes to vasculopathy and renal injury in lupus: in vivo and in vitro evidence. Kidney Int 68: 110–120. 15954900 10.1111/j.1523-1755.2005.00385.x

[38] Kurosawa S, Stearns-Kurosawa DJ, Hidari N, Esmon CT (1997) Identification of functional endothelial protein C receptor in human plasma. J Clin Invest 100: 411–418. 9218519 10.1172/JCI119548 PMC508205

[39] (2010) U.S. Renal Data System, USRDS 2010 Annual Data Report: Atlas of Chronic Kidney Disease and End-Stage Renal Disease in the United States.

[40] Racusen LC, Solez K, Colvin RB, Bonsib SM, Castro MC, et al (1999) The Banff 97 working classification of renal allograft pathology. Kidney Int 55: 713–723. 9987096 10.1046/j.1523-1755.1999.00299.x

[41] Solez K, Colvin RB, Racusen LC, Haas M, Sis B, et al (2008) Banff 07 classification of renal allograft pathology: updates and future directions. Am J Transplant 8: 753–760. 18294345 10.1111/j.1600-6143.2008.02159.x

[42] Rowshani AT, Scholten EM, Bemelman F, Eikmans M, Idu M, et al (2006) No difference in degree of interstitial Sirius red-stained area in serial biopsies from area under concentration-over-time curves-guided cyclosporine versus tacrolimus-treated renal transplant recipients at one year. J Am Soc Nephrol 17: 305–312. 16306168 10.1681/ASN.2005030249

[43] Scheepstra C, Bemelman FJ, van der Loos C, Rowshani AT, van Donselaar-Van der Pant KA, et al (2008) B cells in cluster or in a scattered pattern do not correlate with clinical outcome of renal allograft rejection. Transplantation 86: 772–778. 18813100 10.1097/TP.0b013e3181860a74

[44] Levey AS, Stevens LA, Schmid CH, Zhang YL, Castro AF, 3rd, et al (2009) A new equation to estimate glomerular filtration rate. Ann Intern Med 150: 604–612. 19414839 10.7326/0003-4819-150-9-200905050-00006 PMC2763564

